# Association between serum complements and kidney function in patients with diabetic kidney disease

**DOI:** 10.3389/fendo.2023.1195966

**Published:** 2023-11-17

**Authors:** Meng-chao Liu, Jia-lin Li, Yue-fen Wang, Yuan Meng, Gui-min Zheng, Zhen Cai, Cun Shen, Meng-di Wang, Xiang-gang Zhu, Yang-zi Chen, Yu-lin Wang, Wen-jing Zhao, Wen-quan Niu, Yao-xian Wang

**Affiliations:** ^1^ The First Clinical Medical College, Beijing University of Chinese Medicine, Beijing, China; ^2^ Department of Nephropathy, Beijing Hospital of Traditional Chinese Medicine, Capital Medical University, Beijing, China; ^3^ Center for Evidence-Based Medicine, Capital Institute of Pediatrics, Beijing, China; ^4^ Henan University of Chinese Medicine, Zhengzhou, China

**Keywords:** serum complement, complement C3, complement C4, diabetic kidney disease, diabetes mellitus, nomogram

## Abstract

**Objective:**

We aimed to explore the association between serum complements and kidney function of diabetic kidney disease (DKD) in Chinese patients.

**Methods:**

This is a retrospective study involving 2,441 participants. DKD was diagnosed according to the Kidney Disease: Improving Global Outcomes (KDIGO) categories. Participants were classified as stages G1-G5 by KDIGO glomerular filtration rate (GFR) categories. Effect sizes are expressed as odds ratio (OR) with 95% confidence interval (CI).

**Results:**

After balancing age, gender, systolic blood pressure (SBP), hemoglobin A1c (HbA1C), serum triglyceride (TG), and urinary albumin-to-creatinine ratio (UACR) between the G2-G5 and control groups, per 0.1 g/L increment in serum complement C3 was significantly associated with a 27.8% reduced risk of DKD at G5 stage (OR, 95% CI, P: 0.722, 0.616-0.847, <0.001) relative to the G1 stage. Conversely, per 0.1 g/L increment in serum complement C4 was associated with an 83.0-177.6% increased risk of G2-G5 stage (P<0.001). Serum complement C1q was not statistically significant compared to controls at all stages prior to or after propensity score matching.

**Conclusions:**

Our results indicate that high concentrations of serum C4 were associated with the significantly elevated risk of kidney function deterioration across all stages, and reduced serum C3 levels with an increased risk of DKD stage G5.

## Introduction

Due to the high prevalence of diabetes, diabetic kidney disease (DKD) has drawn growing concerns, as it emerges as the foremost cause of end-stage kidney disease (ESKD) (1, 2). Despite significant progress in preventive care for diabetic individuals, the incidence of ESKD decreased at the slowest rate compared to complications such as lower limb amputation, acute myocardial infarction, stroke, and hyperglycemic crisis. It is worth noting that the number of deaths attributed to DKD increased by 94% (3). These observations underscore the urgent need to develop novel biomarkers capable of identifying individuals at high risk of deteriorating kidney function in DKD. Further, there is a pressing need to explore new agents that can directly target the pathways that contribute to the development of DKD.

It is widely recognized that various factors are involved in the onset and progression of DKD, but precise mechanisms underlying this disease have not been fully elucidated. It is a challenging task when diabetic individuals progress to the moderate or late stage of DKD. The complement system is a fundamental component of innate immunity and is involved in adaptive immunity, contributing to the clearance of immune complexes, cellular debris, and apoptotic cells. Nevertheless, inadequate regulation or excessive activation of the complement system can create a vicious cycle between the complement system, inflammatory cells, and tissue damage (4). Previous studies have mainly focused on the relationship between nephropathy and complements in membranous nephropathy (MN), IgA nephropathy, membranous proliferative glomerulonephritis (MPGN), and other conditions, and studies examining the involvement of the complement system in DKD have emerged in recent years, with compelling evidence demonstrating the activation of the complement system in DKD (5–9). While serum complements C3, C4, and C1q are widely available in clinical practice, studies examining the relationship between serum complements and kidney function in DKD patients are sparse in the literature and inadequate in sample sizes. To the best of our knowledge, this is the first study to simultaneously explore the association between three serum complements (C3, C4, and C1q) and kidney function in DKD patients. Therefore, to enhance clinical application, we examined the relationship between kidney function and serum complement levels in 2,441 patients with DKD and established a nomogram that can be applicable in clinical practice.

## Methods

### Study design

This retrospective study was approved by the Ethics Committee of Beijing Hospital of Traditional Chinese Medicine, Capital Medical University (2020BL02-062), in accordance with the principles of the Helsinki Declaration. As this study was retrospective in nature and all participants’ information was anonymized and de-identified prior to analysis, and the need for written informed consent was waived by the Committee.

### Inclusion criteria

All study participants, ranging in age from 18 to 80 years, were hospitalized for DKD at the Beijing Hospital of Traditional Chinese Medicine Affiliated to Capital Medical University from January 2011 to January 2021.

### Exclusion criteria

Initially, 4,903 inpatients with DKD were included. Among these, 2,462 patients were excluded for the following reasons: 1) undergoing dialysis or kidney transplantation; 2) having a definitive diagnosis of non-DKD; 3) experiencing diabetic ketoacidosis, hyperosmolar hyperglycemia coma, or other acute diabetic complications; 4) being complicated with severe primary diseases of the respiratory, digestive, blood systems, or other serious infections, or being found to have malignant tumors; 5) lacking necessary information, including serum creatinine, urinary albumin to creatinine ratio (UACR), and serum complement (C3, C4, C1q).

Ultimately, total 2,441 eligible patients with DKD were enrolled in the analysis, comprising 1,471 men and 970 women. Of these, 821 patients with an estimated glomerular filtration rate (eGFR) > 90 ml/min/1.73m^2^ were designated as the control group, while the remaining patients were classified as the case group.

### Diagnosis of DKD

The 2012 KDIGO clinical practice guidelines provide a clear definition of DKD, which is characterized by an UACR ≥30 mg/g, or an eGFR <60 mL/min/1.73 m^2^ in the absence of signs or symptoms indicating other primary causes of kidney damage (10). The eGFR was calculated using the 2009 CKD Epidemiology Collaboration (CKD-EPI) equation (11).

### Stages of DKD

The KDIGO risk categories comprise two primary components: persistent albuminuria and GFR. The GFR categories are classified as follows: G1 (GFR in ml/min/1.73m^2^: ≥ 90), G2 (60-89), G3 (45-59), G3b (30-44), G4 (15-29), and G5 (≤ 15 or treatment by dialysis). Due to limited sample sizes, the G3a and G3b categories were combined into a single category, namely G3. The persistent albuminuria categories are classified as A1 (UACR in mg/g: ≤ 30), A2 (30-299), and A3 (≥ 300) (10). In the present study, patients with DKD were categorized into five stages based on the KDIGO GFR categories, including G1 (n=821), G2 (n=517), G3 (n=558), G4 (n=258), and G5 (n=287).

### Clinical and biochemical indices

Data utilized in this study were extracted from the scientific research sharing platform (Yidu Cloud Research Collaboration Platform) of Beijing Hospital of Traditional Chinese Medicine affiliated to Capital Medical University. Standardized questionnaires were administered to all study participants to collect demographics and medical histories.

The definition of diabetes mellitus was based on the American Diabetes Association’s Classification and Diagnosis of Diabetes: Standards of Medical Care in Diabetes-2021 (12). Hypertension was defined as systolic blood pressure (SBP) of 140 mmHg or greater, diastolic blood pressure (DBP) of 90 mmHg or greater, or use of antihypertensive medication (13).

Laboratory tests were conducted on serum samples obtained by venipuncture from patients after an 8-hour fast. Serum was separated immediately after centrifugation within 1 hour of blood sampling. Serum complements were measured using immunotransmission turbidimetry, including serum C3, C4, and C1q. The reference ranges for serum C3, C4, and C1q were 0.75-1.40 (g/L), 0.10-0.40 (g/L), and 159.0-233.0 mg/L, respectively.

Creatinine concentrations were determined using the enzymatic method, while urine microalbumin was determined using the immunoturbidimetric method. Serum triglycerides (TG), total cholesterol (TC), high-density lipoprotein cholesterol (HDL-C), and low-density lipoprotein cholesterol (LDL-C) concentrations were measured using an automated biochemical analyzer. HbA1c was determined using high-performance liquid chromatography.

All tests were conducted twice before reporting and were performed by trained laboratory staff at the Beijing Hospital of Traditional Chinese Medicine, Capital Medical University.

### Statistical analysis

All statistical analyses were performed using STATA version 16 (StataCorp, College Station, TX, USA). Normally distributed continuous variables are presented as mean (standard deviation), skewed continuous variables as median (interquartile range), and categorical variables as count (percent). Differences between groups were evaluated using the χ^2^ test for categorical variables and the t test or Wilcoxon rank sum test for continuous variables. The association between complements and stages of kidney function in diabetes mellitus, before and after adjusting for confounders, was analyzed using logistic regression analysis, with effect sizes reported as odds ratio (OR) with 95% confidence interval (95% CI). To control for potential bias in group-based equivalents, propensity score matching (PSM) was employed. Age, gender, SBP, HbA1C, TG, and UACR were *a priori* balanced in PSM analysis. Age, SBP and UACR differed significantly among groups in unadjusted model. As reported, DKD progression differed in men and women (14). It is well-established that optimal glycemic control can reduce the risk of DKD progression (15, 16). Furthermore, published literature supports an association between TG levels and DKD progression (17, 18). The calibration was evaluated using the Akaike Information Criterion (AIC), Bayesian Information Criterion (BIC), and -2 log likelihood ratio tests. The discrimination was assessed by net reclassification improvement (NRI) and integrated differential improvement (IDI). A nomogram was constructed using the “RMS” package in the R programming environment (version 3.5.2). The statistical significance was set at a p-value of less than 0.05.

## Results

### Baseline characteristics

**Table 1** presents the baseline characteristics of the study participants. The G2-G5 stages were observed to be older and had higher SBP and lower HbA1C levels compared to the G1 stage. Serum C3 was significantly lower in G3-G5 stages compared to the control group (P<0.01), whereas serum C4 levels were significantly higher in G2-G5 compared to the G1 group (P<0.01). However, no statistically significant difference was observed in serum C1q levels at any stage (P>0.05).

**Table 1:** Baseline characteristics of study participants in this study.

Characteristics	Patients with DKD				
	G1	G2	G3	G4	G5
Sample size	821	517	558	258	287
Age(years)	57 (50-63)	66 (58-74)**	68 (59-76)**	64 (56-74)**	62 (54-69)**
Male(n,%)	477, 58.10	331, 64.00*	329, 59.00	150, 58.10	184, 64.10
SBP(mmHg)	131 (120-145)	140 (128-150)**	140 (130-158)**	150 (135-161)**	156 (140-170)**
DBP(mmHg)	80 (74-89)	80 (70-89)	80 (70-90)	80 (70-88)	81 (75-90)**
HbA1c(%)	7.80 (6.60-9.60)	7.20 (6.40-8.70)**	7.00 (6.30-8.10)**	6.60 (6.00-7.65)**	6.20 (5.70-6.70)**
TG(mmol/L)	1.61 (1.14-2.37)	1.60 (1.16-2.24)	1.60 (1.14-2.41)	1.74 (1.25-2.41)	1.64 (1.14-2.31)
TC(mmol/L)	4.66 (3.96-5.39)	4.62 (3.89-5.48)	4.67 (3.78-5.68)	4.93 (3.97-6.14)**	4.69 (3.77-5.45)
LDL-C(mmol/L)	2.64 (2.12-3.25)	2.68 (2.09-3.38)	2.69 (2.03-3.42)	2.84 (2.11-3.61)**	2.68 (2.08-3.33)
HDL-C(mmol/L)	1.17 (0.99-1.35)	1.12 (0.97-1.35)	1.17 (0.97-1.38)	1.15 (0.97-1.38)	1.10 (0.95-1.33)*
eGFR(ml/min/1.73m²)	103.16 (96.94-111.79)	76.36 (67.84-83.37)**	45.72 (38.63-53.11)**	21.60 (18.54-25.66)**	9.47 (7.28-11.55)**
UACR(mg/g)	37.81 (34.14-193.30)	157.19 (37.11-952.50)**	506.88 (61.22-2084.66)**	2159.22 (770.02-4254.55)**	2958.48 (1518.14-4930.85)**
C3(g/L)	1.10 (0.95-1.24)	1.07 (0.95-1.20)	1.04 (0.91-1.18)**	0.99 (0.86-1.13)**	0.90 (0.78-1.04)**
C4(g/L)	0.22 (0.18-0.26)	0.23 (0.19-0.28)**	0.24 (0.20-0.29)**	0.25 (0.20-0.30)**	0.24 (0.20-0.29)**
C1q(mg/L)	175.30 (158.28-206.10)	181.95 (159.29-208.14)	183.43 (157.00-210.48)	179.60 (159.20-212.50)	179.22 (158.22-210.20)

SBP, systolic blood pressure; DBP, diastolic blood pressure; HbA1c, hemoglobin A1c; TG, serum triglyceride; TC, total cholesterol; LDL-C, low-density lipoprotein cholesterol; HDL-C, high-density lipoprotein cholesterol; eGFR, estimated glomerular filtration rate; UACR, urinary albumin-to-creatinine ratio; C3, Complement 3; C4, Complement 4; C1q, Complement 1q. Continuous variables are expressed as median (interquartile range), and categorical variables as count (percent). Between-group comparison was done using Wilcoxon rank sum test or χ^2^ test, where appropriate. *P<0.05; **P< 0.01.

### Serum complements and DKD stages

**Table 2** displays the correlation between serum C3, C4, C1q at G2-G5 stages in patients with type 2 diabetes before and after PSM. Using the Bonferroni correction, a P-value less than 0.05/12 was considered significant. After balancing covariates including age, gender, SBP, HbA1C, TG, and UACR between the G2-G5 and control groups, per 0.1 g/L increment in serum C3 was significantly associated with a 27.8% reduced risk of DKD at G5 stage (OR, 95% CI, P: 0.722, 0.616-0.847, <0.001) relative to the G1 stage. Conversely, per 0.1 g/L increment in serum C4 was associated with an 83.0-177.6% increased risk of G2-G5 stage (P<0.001). Serum C1q was not statistically significant at all stages before or after PSM.

**Table 2:** Risk prediction of complements for DKD at different stages.

Significant risk factors	G1	G 2	G 3	G 4	G 5
C3 (+0.1 g/L)	Ref.	0.956, 0.910 to 1.003, p=0.068	0.885, 0.842 to 0.931, p<0.001	0.822, 0.767 to 0.880, p<0.001	0.646, 0.598 to 0.698, P<0.001
C4 (+0.1 g/L)	Ref.	1.323,1.132 to 1.546, P<0.001	1.627, 1.395 to 1.897, p<0.001	1.839, 1.521 to 2.224, p<0.001	1.689, 1.407 to 2.027, P<0.001
C1q (+10mg/L)	Ref.	1.044, 1.000 to 1.090, P=0.049	1.039, 0.998 to 1.082, p=0.063	1.041, 0.987 to 1.098, P=0.142	1.030, 0.978 to 1.084, P=0.261
After propensity score matching for age, gender, SBP, HbA1C, TG and ACR					
C3 (+0.1 g/L)	Ref.	1.079, 1.000 to 1.160, P=0.038	0.960, 0.885 to 1.041, P=0.322	0.911, 0.813 to 1.021, P=0.110	0.722, 0.616 to 0.847, P<0.001
C4 (+0.1 g/L)	Ref.	1.830, 1.436 to 2.332, P<0.001	1.860, 1.433 to 2.413, P<0.001	1.879, 1.291 to 2.735, P=0.001	2.776, 1.670 to 4.610, P<0.001
C1q (+10 mg/L)	Ref.	1.038, 0.973 to 1.107, P=0.256	1.100, 1.022 to 1.183, P=0.011	1.101, 0.997 to 1.216, P=0.058	1.123, 0.973 to 1.296, P=0.111

C3, Complement 3; C4, Complement 4; C1q, Complement 1q. Data are expressed as odds ratio, 95% confidence interval, P value.

### Prediction accuracy assessment

**Table 3** illustrates the prediction accuracy achieved by separately adding serum complements to the basic model (including age, gender, SBP, HbA1C,TG and UACR). Using logistic regression analysis after PSM, only C4 was added to the basic model in G2-G4 stages, whereas both C3 and C4 were added to the basic model in G5 stage. Calibration analysis showed that the addition of complements to the basic model resulted in a reduction in both AIC and BIC statistics by more than 10 for each stage, and the likelihood ratio test indicated a statistically significant difference with added complements for all stages. Discrimination analysis using both NRI and IDI showed a significant improvement in prediction accuracy upon the addition (19).

**Table 3:** Prediction accuracy gained by adding complement to basic model for DKD at different stages.

Statistics	G 2		G 3		G 4		G5	
	Basic model	Basic model plus C4	Basic model	Basic model plus C4	Basic model	Basic model plus C4	Basic model	Basic model plus C3 and C4
Calibration								
AIC	1310.33	1283.65	1168.43	1133.27	666.14	637.32	490.46	404.77
BIC	1345.79	1324.18	1204.17	1174.11	700.28	676.34	524.81	448.93
LR test (χ2)	Ref.	28.68	Ref.	37.16	Ref.	30.82	Ref.	89.70
LR test P value	Ref.	<0.001	Ref.	<0.001	Ref.	<0.001	Ref.	<0.001
Discrimination								
NRI (P value)	Ref.	<0.001	Ref.	0.007	Ref.	<0.001	Ref.	<0.001
IDI (P value)	Ref.	<0.001	Ref.	<0.001	Ref.	<0.001	Ref.	<0.001

AIC, Akaike information criterion; BIC, Bayesian information criterion; LR, likelihood ratio; NRI net reclassification improvement; IDI, integrated discrimination improvement; C3, Complement 3; C4, Complement 4.

### Interaction of serum complements

**Table 4** presents the effect-size estimates for the interaction between complement and DKD kidney function. To synthesize 12 new variables, we binarized C3, C4, and C1q based on the median values of all study participants and divided them into high and low groups. To prevent false positives, we considered P<0.05/12 statistically significant according to the Bonferroni correction. According to the results, the combination of low C3 and high C4 demonstrated the maximum effect-size estimate in the G4-G5 stage (OR=7.412 and 9.973, 95%CL: 3.708-14.818 and 4.727-21.041, P<0.001).

**Table 4:** The effect-size estimates for the interaction between complement and DKD GFR categories.

Interaction items		OR, 95% CI, P			
		G2	G3	G4	G5
C3–C4	High C3– Low C4	Ref.	Ref.	Ref.	Ref.
	Low C3– High C4	1.488, 0.930 to 2.380, P=0.097	2.896, 1.809 to 4.602, P<0.001	7.412, 3.708 to 14.818, P<0.001	9.973, 4.727 to 21.041, P<0.001
	High C3– High C4	1.417, 0.994 to 2.022, P=0.054	1.354, 0.926 to 1.980, P=0.118	3.141, 1.648 to 5.986, P=0.001	1.758, 1.077 to 4.287, P=0.030
	Low C3– Low C4	0.795, 0.561 to 1.127, P=0.197	0.949, 0.653 to 1.380, P=0.786	2.610, 1.406 to 4.845, P=0.002	2.198, 1.227 to 3.940, P=0.008
C4–C1q	Low C4– Low C1q	Ref.	Ref.	Ref.	Ref.
	High C4– Low C1q	1.544, 0.788 to 3.028, P=0.206	2.400, 1.285 to 4.483, P=0.06	1.831, 0.801 to 4.185, P=0.152	4.000, 1.488 to 10.755, P=0.006
	High C4– High C1q	2.473, 1.406 to 4.350, P=0.002	3.482, 1,872 to 6.474, P<0.001	2.096, 0.999 to 4.396, P=0.050	2.210, 0.818 to 5.971, P=0.118
	Low C4– High C1q	1.245, 0.696 to 2.227, P=0.459	1.703, 0.933 to 3.110, P=0.003	0.787, 0.295 to 2.099, P=0.632	1.367, 0.510 to 3.666, P=0.535
C3–C1q	High C3– Low C1q	Ref.	Ref.	Ref.	Ref.
	Low C3– High C1q	1.837, 0.983 to 3.433, P=0.057	2.206, 1.041 to 4.064, p=0.038	2.547, 1.022 to 6.348, P=0.045	4.314, 1.243 to 14.973, P=0.021
	High C3– High C1q	1.041, 0.577 to 1.877, P=0.894	1.345, 0.760 to 2386, p=0.309	1.238, 0.493 to 3.110, P=0.649	0.349, 0.072 to 1.690, P=0.494
	Low C3– Low C1q	0.679, 0.387 to 1.193 P=0.179	0.758, 0.433 to 1.330, P=0.335	1.775, 0.807 to 3.901, P=0.153	1.447, 0.476 to 4.402, P=0.515

C3, Complement 3; C4, Complement 4; C1q, Complement 1q. Data are expressed as odds ratio, 95% confidence interval, P value.

### Nomogram prediction model

**Figure 1:**
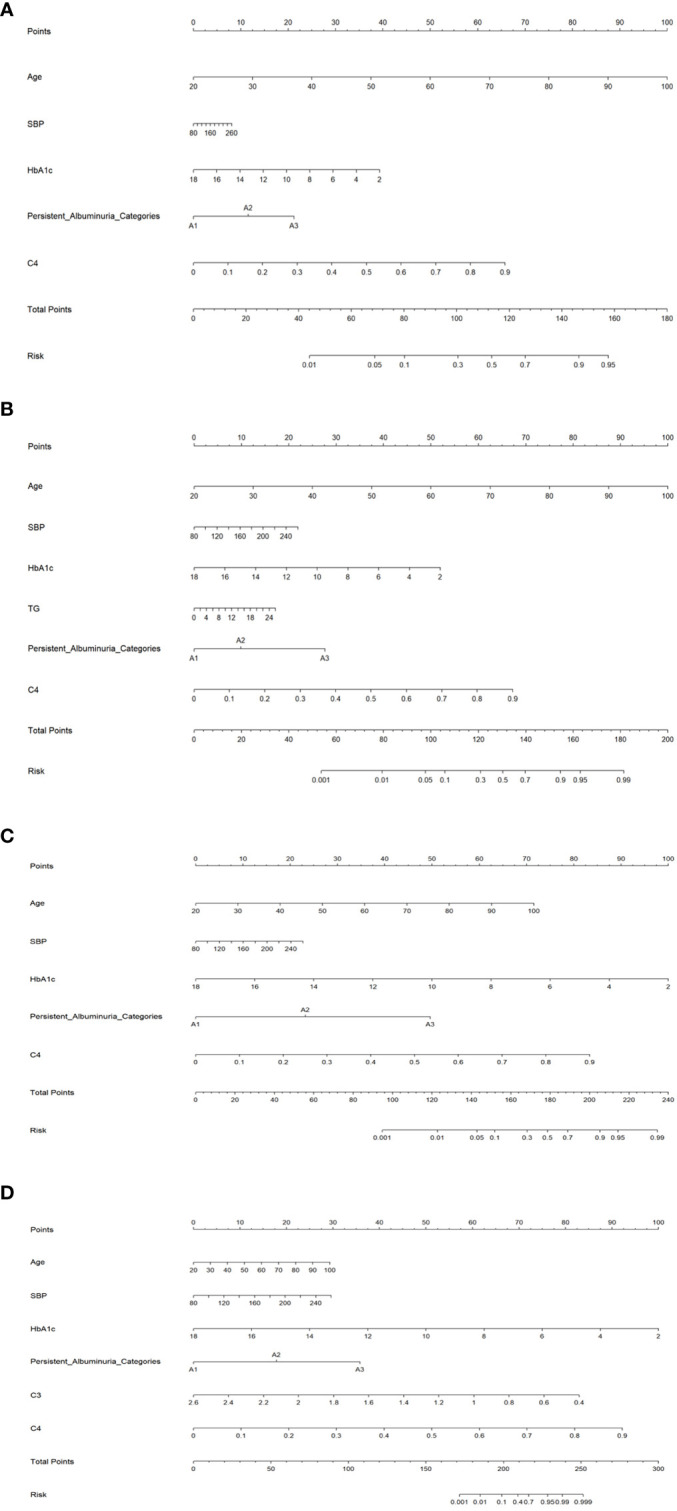
Nomogram prediction models for the G2 **(A)**, G3 **(B)**, G4 **(C)**, and G5 **(D)** stages. SBP, systolic blood pressure; HbA1c, hemoglobin A1c; TG, serum triglyceride; C3, Complement 3; C4, Complement 4.

To facilitate practical applications, we developed a nomogram prediction model for the DKD stages G2-G5, as depicted in **Figure 1**. Significant factors in the nomogram model were selected by forward logistic regression analyses at a significance level of 0.4%. The accuracy of the four nomogram models was satisfactory, with the C index values of 0.784, 0.876, 0.922, and 0.977 (all P<0.001).

## Discussion

The primary aim of this study was to investigate the relationship between serum complements and kidney function levels in patients with DKD. To our knowledge, this was the first study that has explored this relation using the KDIGO GFR categories. Our results indicate that high concentrations of serum C4 were associated with the significantly elevated risk of kidney function deterioration across all stages. Whereas reduced serum C3 levels were associated to an increased risk of DKD stage G5. These results suggest that serum complements may serve as valuable indicators for worsening kidney function in patients with type 2 diabetes.

A substantial body of evidence supports the involvement of the complement system in DKD progression. Among these components, C3, a crucial element of the three complement activation pathways, is well evidenced in support of its contributing role. C3 deposits in the glomeruli of DN mice from early stages, and levels of C3a and the C3a receptor (C3aR) are elevated (20). The inflammatory response and T cell adaptive immunity in C3aR knockout mice are significantly suppressed, which alleviates diabetic kidney injury (20). Treatment of DN mice with C3aR antagonists increases podocyte density and maintains their phenotype, thereby limiting proteinuria and glomerular damage (21). Patients with C3 deposition exhibit higher interstitial fibrosis and tubular atrophy (IFTA) scores and a greater proportion of global sclerosis compared to those without C3 deposition, and complement deposition incidence is higher in advanced DN than in early DN (20, 22). Additionally, it has been discovered that C3a and C5a receptor antagonists can alleviate glomerular fibrosis in DKD patients by improving the endothelial-myofibroblast transition by inhibiting the Wnt/β-catenin signaling pathway (23). C4d deposits in both glomeruli and arterioles, and C1q deposits in both glomerular hili and arterioles are increased compared to the non-DN group. Among them, glomerular C4d deposition correlates with DN severity, while others do not (24). Furthermore, patients with C4c plus C3c and C1q deposits experience heavier proteinuria than those with C4c plus only one of the deposits or those with DN without C4c deposits (25). These studies collectively suggest that the complement system is implicated in the development of renal pathologies in diabetic patients and is a promising target for inhibiting DKD progression.

Several studies have explored the correlation between serum complement and DKD. A recent study found that decreased serum C3 levels and increased serum C4 levels are significantly associated with more severe kidney dysfunction and worse renal outcomes in DN patients (26), which aligns with our findings. However, a relevant exploration of serum C1q was not performed in this study. While there was no significant difference in serum C4 between DN patients with and without C4c deposition, and the median GFR of the higher serum C4 group was lower, but the difference in GFR between the two groups did not achieve statistical significance, possibly due to the insufficient sample size (25). Regarding the plausible mechanisms of serum complements in patients with DKD, many publications related to various forms of renal dysfunction reported that circulatory C3 level decreased and renal tissue deposition of C3 increased, which may be related to abnormal activation of complement C3 leading to the excessive consumption and cleavage of C3 (26–29). Interestingly, it has been shown that not all DN patients had C3 deposition and that C3 deposition was significantly more in late DN than early DN, suggesting that complement activation may be an aggravating factor rather than a causative factor (27, 29). In our study, low serum C3 had the most significant association with the G5 stage of DKD, supporting the above argument. While that the increment in serum C4 may be affected by other factors than renal C4 deposition, such as widespread micro-inflammation in patients with DKD (27). However, the involvement of C1q may be relatively small.

Another notable finding of this study is that serum complements may interact with each other to increase the risk of renal deterioration in DKD, particularly in DKD stages 4-5, where low serum C3 and high serum C4 demonstrate a significant synergistic interaction, far exceeding other combinations. We must acknowledge that our findings are preliminary, and the complex intrinsic associations between complements warrant further exploration.

To facilitate clinical application, we developed a nomogram prediction model for kidney function grading in DKD based on statistical significance and conventional attributes. Importantly, the model exhibits decent prediction accuracy, which can assist in clinical decision-making and personalized management of DKD.

It is worth mentioning that patients with advanced DKD often show heterogeneity in glycemic control. In our study, HbA1c in the G5 group of DKD was significantly better than that in the G3 and G4 groups, and the main reason may be that the low GFR of DKD patients in the G5 group led to the decreased ability of the kidneys to expel insulin and hypoglycemic drugs, and the decrease of renal gluconogenesis (30, 31). Also, assessing HbA1c control in patients with advanced DKD may be biased by abnormalities in blood haemoglobin (30).

Several limitations of this study must be acknowledged. First, the cross-sectional design constrains the inference of any temporal relationship and cannot be used to establish a causal relationship between serum complements and kidney function in DKD. Second, all study participants were recruited from a single center, which may have led to population stratification and necessitates additional external validation. Furthermore, certain participant characteristics, such as obesity and insulin secretion levels, were not available, which may confound the association.

## Conclusion

Despite these limitations, our study, with its large sample size, indicates that serum C4 can serve as a biomarker of kidney function deterioration in DKD, serum C3 as a biomarker of advanced DKD, and that there may be a synergistic effect between serum C4 and C3 in worsening kidney function in DKD. At present, numerous studies are being conducted on inhibitors of complement components (such as C1, C3, C5, and C5aR1), and the inhibition of pathological complement activation has emerged as a potential therapy to prevent DKD progression. Therefore, we establish background data to further investigate the role of complements in the pathogenesis of DKD and to explore their potential as an adjuvant therapy to control DKD progression.

## Data availability statement

The raw data supporting the conclusions of this article will be made available by the authors, without undue reservation.

## Ethics statement

The studies involving humans were approved by The Ethics Committee of Beijing Hospital of Traditional Chinese Medicine, Capital Medical University. The studies were conducted in accordance with the local legislation and institutional requirements. The ethics committee/institutional review board waived the requirement of written informed consent for participation from the participants or the participants’ legal guardians/next of kin because This study was retrospective in nature and all participants’ information was anonymized and de-identified prior to analysis.

## Author contributions

Y-XW and W-JZ planned and designed the study. W-QN directed its implementation and drafted the protocol. Y-FW, YM, G-MZ, ZC, CS, X-GZ, Y-ZC, and M-DW contributed to data acquisition. Y-FW, M-CL and Y-LW obtained statutory and ethics approvals. M-CL and J-LL conducted statistical analyses. W-JZ, W-QN and Y-XW did the quality control. M-CL and J-LL wrote the manuscript. All authors contributed to the article and approved the submitted version.
